# Leukocyte margination at arteriole shear rate

**DOI:** 10.14814/phy2.12037

**Published:** 2014-06-11

**Authors:** Naoki Takeishi, Yohsuke Imai, Keita Nakaaki, Takami Yamaguchi, Takuji Ishikawa

**Affiliations:** 1 Department of Biomedical Engineering, Tohoku University, AobaSendai, Japan; 2 Department of Bioengineering and Robotics, Tohoku University, AobaSendai, Japan

**Keywords:** Computational biomechanics, leukocyte margination, microcirculation

## Abstract

We numerically investigated margination of leukocytes at arteriole shear rate in straight circular channels with diameters ranging from 10 to 22 *μ*m. Our results demonstrated that passing motion of RBCs effectively induces leukocyte margination not only in small channels but also in large channels. A longer time is needed for margination to occur in a larger channel, but once a leukocyte has marginated, passing motion of RBCs occurs continuously independent of the channel diameter, and leukocyte margination is sustained for a long duration. We also show that leukocytes rarely approach the wall surface to within a microvillus length at arteriole shear rate.

## Abstract (precis)

We numerically investigated margination of leukocytes at arteriole shear rate in straight circular channels with diameters ranging from 10 to 22 *µ*m. Our results demonstrated that passing motion of RBCs effectively induces leukocyte margination not only in small channels but also in large channels. We also show that leukocytes rarely approach the wall surface to within a microvillus length at arteriole shear rate.

## Introduction

Because of lift forces perpendicular to the wall, induced by the wall and share gradient, a deformable particle flowing in a channel migrates to the center of the channel. Therefore, in microcirculation, red blood cells (RBCs) exhibit axial migration and generate a cell‐depleted peripheral layer (CDPL). Leukocytes, in contrast to RBCs, appear to flow primarily in the peripheral layer. This is termed margination, which is the first step in the firm adhesion of leukocytes to the endothelium. Although the deformability of leukocytes is much smaller than RBCs, lift forces are still present even for leukocytes. Schmid‐Schönbein et al. (1980) proposed one possible mechanism for leukocyte margination. They observed that RBCs passed a leukocyte in venules of diameter 10 *μ*m or larger, and concluded that the passing motion of RBCs provides forces toward the wall. In contrast, experimental studies using relatively large vessels (Goldsmith and Spain 1984; Nobis et al. 1985) concluded that aggregation of RBCs is necessary for leukocyte margination in such large vessels. However, recent numerical studies using two‐dimensional models showed that RBC aggregation is not a necessary condition for margination (Freund 2007), and that aggregation enhances leukocyte margination (Fedosov et al. 2012).

The passing motion of RBCs is a result of the velocity difference between RBCs flowing near the center of the vessel and leukocytes flowing near the wall. Once a leukocyte is marginated, passing motion will occur independent of the venule diameter and provide forces toward the wall to sustain leukocyte margination. Such passing motion will also occur in arterioles. Previous studies have shown that leukocyte rolling and adhesion are often found in venules but rarely in arterioles (House and Lipowsky 1987, 1991; Ley and Gaehtgens 1991; Perry and Granger 1991; Nazziola and House 1992). However, it remains unclear whether leukocytes fail to marginate, contact endothelial cells, or roll on endothelial cells in arterioles.

In this study, we numerically investigated the flow of leukocytes and RBCs in straight channels for a number of channel diameters at arteriole shear rate. We show that leukocyte margination occurs due to the passing motion of RBCs for a wide range of channel diameters. We also show that the separation distance between the marginated leukocyte and wall surface depends on shear rate, and that leukocytes rarely approach the wall surface to within a microvillus length at arteriole shear rate.

## Methods

### Numerical model

We take blood to consist of plasma, RBCs, and leukocytes. Hereafter, the superscripts *R* and *L* represent parameters for RBCs and leukocytes, respectively. An RBC is modeled as a biconcave capsule, with a Newtonian fluid enclosed by a thin elastic membrane. The diameter of an RBC is *d*^*R *^= 8 *μ*m, and the thickness is approximately 2 *μ*m. The membrane follows the constitutive law proposed by Skalak et al. (1973), where the membrane elasticity is characterized by a surface shear elastic modulus *G*_*s*_ and area dilation modulus *K*_*s*_ = *G*_*s*_(1 + 2*C*). The surface shear modulus was determined to be 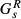 = 4.0 × 10^−6^ N/m by a numerical test reproducing the stretching of an RBC by optical tweezers (Suresh et al. 2005). The area dilation modulus was set to *C*^*R*^ = 10^2^ to express the nearly incompressible property of the RBC membrane. Additionally, the membrane is considered to have nonzero bending resistance (Li et al. 2005) with a bending modulus 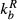 = 5.8 × 10^−19^ N m (Puig‐de‐Morales‐Marinkovic et al. 2007). The viscosity of cytoplasm is taken to be *μ*^*R*^ = 6.0 × 10^−3 ^Pa s, which is five times higher than the viscosity of plasma (1.2 × 10^−3 ^Pa s).

Ting‐Beall et al. (1993) reported that the volume of neutrophils is approximately 300 *μ*m^3^, corresponding to the diameter of 8.2–8.3 *μ*m. A leukocyte is hence modeled as a spherical capsule of the same diameter as an RBC (*d*^*L*^ = 8 *μ*m), where microvilli on the leukocyte surface are ignored. The deformability of leukocytes is much lower than that of RBCs. Chien et al. (1987) reported that the steady‐state deformation of leukocytes shows that they are only four times stiffer than RBCs, but that the cytoplasm viscosity of leukocytes is 1000 times higher than that of RBCs. In this study, for simplicity, the deformability is controlled by modulating the relative surface shear elastic modulus *R*_Gs_ = 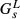/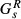. To cover a wide range of deformability, we examined three values of *R*_Gs_ for leukocytes: *R*_Gs_ = 10^1^, 10^2^, and 10^3^. The other leukocyte parameters, *C*^*L*^, 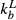, and *μ*^*L*^, are the same as an RBC. When a leukocyte approaches to endothelial cells, ligand–receptor interactions would occur in vivo. However, in this study, we concentrate on hydrodynamic processes, and do not model ligand–receptor interactions.

We performed numerical simulations of blood flows in straight circular channels with the diameter *D* ranging from 10 to 22 *μ*m. Koutsiaris et al. (2013) quantified the wall shear rate in human conjunctival precapillary arterioles. They reported that the average wall shear rate is 600–700/s. Hence, in this study, the pressure gradient is given such that the wall shear rate is 670/s, mimicking arterioles. The length of the computational domain *L* is approximately 100 *μ*m, and the periodic boundary condition is employed. One leukocyte is placed in the computational domain, with the number of RBCs depending on hematocrit (Hct).

This is a fluid–membrane interaction problem, where the fluid mechanics of the plasma and cytoplasm is coupled with the solid mechanics of cell membranes. The membrane mechanics is solved by a finite element method (FEM) (Walter et al. 2010) and the fluid mechanics is solved by a lattice‐Boltzmann method (LBM) (Chen and Doolen 1998), where FEM and LBM are coupled by an immersed boundary method (Peskin 2002). A volume‐of‐fluid method (Yokoi 2007) and front‐tracking method (Unverdi and Tryggvason 1992) are also employed to update the value of the viscosity in the fluid mesh. The mesh size of LBM is 0.25 *μ*m, and that of FEM is also approximately 0.25 *μ*m. All the procedures are fully implemented on graphics processing unit (GPU) to accelerate numerical simulation (Miki et al. 2012). We examined the deformation of RBCs in shear flow and the thickness of the CDPL in channel flow. The comparisons of our results with previous experimental and numerical results are presented in the Appendix, in which we validate the current method.

### Analysis

To determine the position of the leukocyte in the numerical simulations, we measured (calculated) a separation distance *δ*, defined by the shortest distance between the leukocyte membrane and the wall surface. We only used data after the thickness of the CDPL reached a plateau (hereafter this time is referred to as *t *=**0) to reduce the influence of the initial conditions. In most cases, the leukocyte had marginated before *t *=**0, and flowed while maintaining the separation distance within the CDPL thickness. Therefore, we defined leukocyte margination by *δ *< *δ*_CDPL_, where *δ*_CDPL_ refers to the time‐averaged CDPL thickness. To quantify the passing motion of RBCs, we also measured the averaged velocity of RBCs relative to the velocity of the leukocyte. When the relative velocity is positive, RBCs move faster than the leukocyte over both spatial and temporal averages, hence the occurrence of continuous passing motion.

## Results

### Flow of leukocytes and RBCs

**Figure 1.**
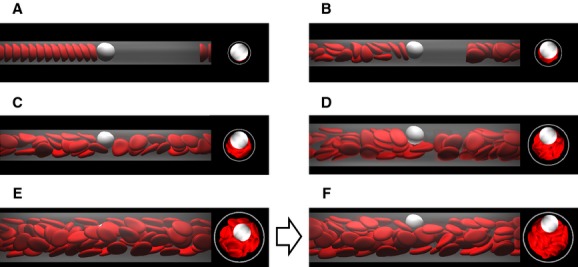
Snapshots of the flow of a leukocyte and RBCs for *R*_Gs_ = 10^2^ and Hct**= 0.2 in channels of diameter *D *=**10 *μ*m (A), 12 *μ*m (B), 14 *μ*m (C), 18 *μ*m (D), 22 *μ*m at *t *=**0 (E), and 22 *μ*m after margination (F). The flow direction is from left to right.

**Figure 2.**
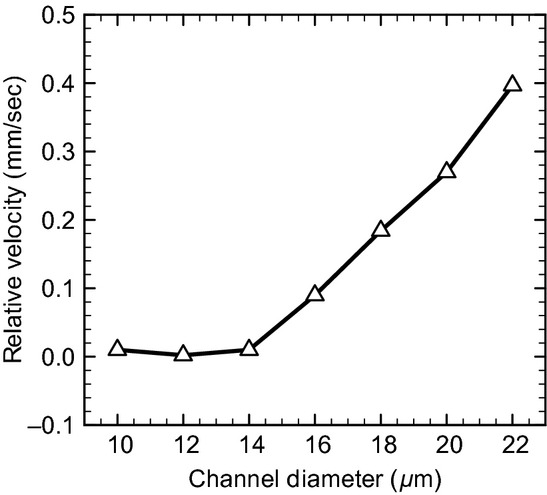
Velocity of RBCs relative to the leukocyte for *R*_Gs_ = 10^2^ and Hct = 0.2.

**Figure 3.**
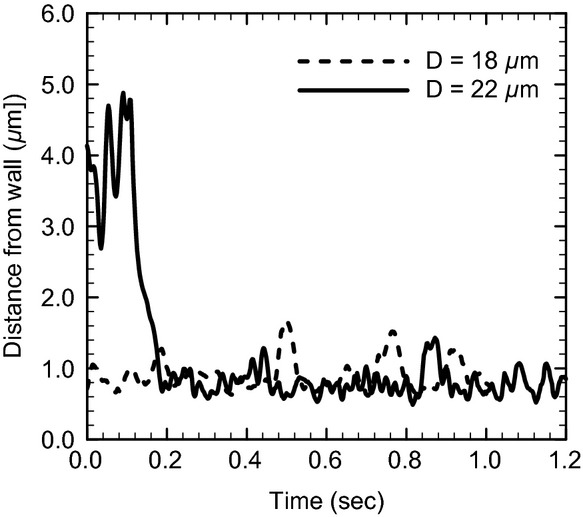
Time histories of the separation distance between the leukocyte membrane and wall surface for *R*_Gs_ = 10^2^ and Hct = 0.2.

First, we show the numerical results of *R*_Gs _= 10^2^ and Hct = 0.2. In a small channel (*D *=**10 *μ*m), the leukocyte and RBCs flowed in the center of the channel and formed a steady, single‐file train, and the RBCs displayed parachute‐shaped deformation (Fig. 1A). When the channel diameter was increased to 12 *μ*m, they still formed a train, but RBCs followed the leukocyte in a multifile motion (Fig. 1B). Because the motion of multifiled RBCs was unsteady, the leukocyte position slightly shifted toward the wall. When the channel diameter was increased further to 14 *μ*m, RBCs started to pass the leukocyte through the gap between the leukocyte and wall (Fig. 1C). The relative velocity of RBCs to the leukocyte then became a positive value, as shown in Figure 2. As the passing motion first occurred, RBCs pushed the leukocyte toward the wall, resulting in leukocyte margination. Even in larger channels (Fig. 1D), the passing motion of RBCs continuously occurred and the marginated leukocyte continued to flow near the wall (Fig. 3). Note that in the case of *D *=**22 *μ*m, the leukocyte membrane was still located completely outside the CDPL at *t *=**0 (Figs. 1E, 3). At this point, the leukocyte was sandwiched by RBCs that were flowing in both the central and peripheral layers. Because of the axial migration of RBCs, the concentration of RBCs was lower in the peripheral layer, and hence the lateral position of the leukocyte gradually moved to the wall. Finally, the leukocyte marginated, and that margination was maintained by the passing motion of RBCs, as observed in smaller channels (Figs. 1F, 3).

**Figure 4.**
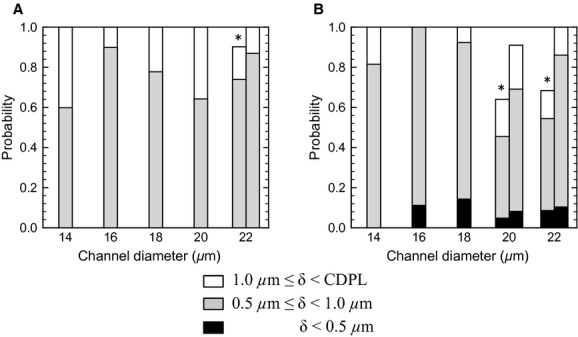
Probability of the separation distance between the leukocyte membrane and wall surface for *R*_Gs_ = 10^2^ and Hct = 0.2 at wall shear rate 670/s (A) and 330/s (B). Probability was calculated by using the data for 0 ≤ *t *≤**1 [s]. Bars with a symbol * indicate that the leukocyte was not fully marginated at *t *=**0. For those cases, probability after margination was also given in the bar to the right. In the case of *D *=**22 *μ*m in (A), for example, this was calculated using the data for 0.2 ≤ *t *≤**1.2 [s] (also see Fig. 3).

The probability of the separation distance is summarized in Figure 4A for *R*_Gs_ = 10^2^ and Hct = 0.2, where the probability was calculated by using data over a time period of 1.0 s from the start of the quasi‐steady state. No significant differences are observed among the channel diameters. The most probable separation distance was between 0.5 and 1.0 *μ*m, smaller than the CDPL thickness (2–3 *μ*m), with a probability of 0.6–0.8. These results show that passing motion induces leukocyte margination effectively for a wide range of the channel diameters. However, we should note that a separation distance less than 0.5 *μ*m did not appear in any cases. To check the effect of numerical resolution, we performed simulations with twice the computational mesh resolution, but no significant differences were found. We also examined the effect of halving the wall shear rate to 330/s, and in this case, the leukocyte membrane sometimes approached to within 0.5 *μ*m of the channel wall, although the probability was still a low value, as shown in Figure 4B.

### Effect of hematocrit and leukocyte deformability

**Figure 5.**
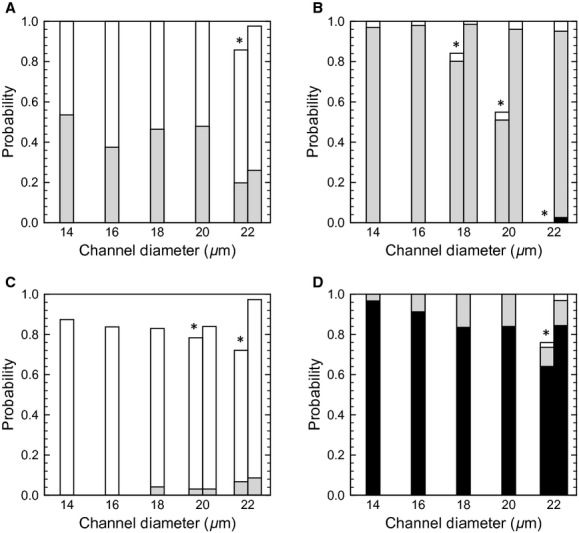
Effect of hematocrit on the separation distance for *R*_Gs_ = 10^2^: Hct = 0.1 (A), and Hct = 0.3 (B). Effect of leukocyte deformability for Hct = 0.2: *R*_Gs _= 10^1^ (C), and *R*_Gs _= 10^3^ (D).

**Figure 6.**
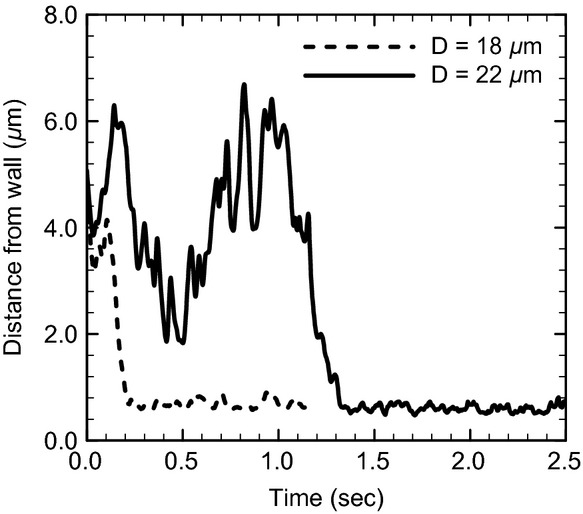
Time histories of the separation distance between the leukocyte membrane and wall surface for *R*_Gs_ = 10^2^ and Hct = 0.3.

The leukocyte position is determined by the balance between the interaction forces from RBCs and the lift force caused by leukocyte deformability. Due to the lift force, the leukocyte moves toward the center of the vessel until the next passing motion occurs. Therefore, we investigated the effect of hematocrit on the separation distance. The most probable distance was not changed significantly by the hematocrit in the range Hct**= 0.1–0.3. In the case of Hct = 0.1 (Fig. 5A), as expected, the probability of *δ *≥ 1.0 *μ*m increased because the leukocyte position fluctuated in time due to the low frequency of interaction with RBCs. By contrast, the leukocyte position was stabilized in the case of Hct = 0.3 (Fig. 5B), but the probability of *δ *≤ 0.5 *μ*m was still nearly zero. Another important aspect of Hct**= 0.3 is that the time required for the initial margination to occur is longer than that at lower Hct. For example, the leukocyte membrane did not reach the CDPL at *t *=**0 even for *D *=**18 *μ*m, as shown in Figure 6.

When the leukocyte is modeled by a rigid sphere, the separation distance will become much smaller because of no lift forces. Figure 5D shows the separation distance for a nearly rigid sphere (*R*_Gs_ = 10^3^) at Hct = 0.2, and the separation distance for *R*_Gs _= 10 is also shown in Figure 5C as a reference. As expected, the surface of the nearly rigid sphere could approach the wall in the range *δ *≤ 0.5 *μ*m.

## Discussion

### Leukocyte margination by passing motion of RBCs

Our results demonstrated that leukocytes are able to marginate in a straight channel in a wide range of diameters because of the passing motion of RBCs. Schmid‐Schönbein et al. (1980) observed that a leukocyte and RBCs formed a single‐file train in 7‐ to 10‐*μ*m venules, and RBCs started to pass the leukocyte when the channel diameter increased to 10 *μ*m or larger. They proposed that this passing motion pushing the leukocyte toward the wall is the mechanism of leukocyte margination. Their observation is consistent with our simulation at an arteriole wall shear rate of 670/s, in which a transition was observed when the size difference between the channel and leukocyte was slightly larger than the thickness of an RBC (*D* – *d*^*L*^ ≈ 4 *μ*m). Nobis et al. (1985) investigated the distribution of leukocytes in plasma, saline, and dextran solutions with Hct = 0.4 using a 69‐*μ*m‐diameter glass capillary, and showed that the highest rate of margination was obtained in the dextran solution, which induces aggregation of RBCs. They concluded that the passing motion of RBCs is only responsible for the mechanism of leukocyte margination in vessels less than 15 *μ*m, and aggregation of RBCs is necessary for leukocyte margination in larger vessels. A similar conclusion was reached by Goldsmith and Spain (1984) who examined leukocyte margination in glass tubes with a diameter larger than 100 *μ*m. However, in our simulation, leukocyte margination occurred without RBC aggregation even in large channels, which has also shown in two‐dimensional numerical simulations (Freund 2007; Fedosov et al. 2012). Although a larger channel requires a longer time until leukocyte margination occurs, particularly in higher hematocrit conditions, once a leukocyte has marginated, passing motion of RBCs continuously occurs independent of the channel diameter, and leukocyte margination is sustained for a long duration (Figs. 3, 6). Our results suggest that the channel length in previous experimental studies may not be long enough to measure leukocyte margination in large channels, and as a result less margination was found in these studies. A recent experimental study by Jain and Munn (2009), in which leukocyte margination was studied using rectangular channels, supports this hypothesis. They observed the motion of cells at points between the inlet and 5 mm downstream of the inlet. They showed that the number of marginated leukocytes in a 25‐*μ*m‐width channel with plasma (Hct**= 0.2) reached a plateau within 5 mm, but the number increased almost linearly with the distance in channels with widths of 50 *μ*m or 75 *μ*m.

### Leukocytes rarely approach the wall surface within microvilli length

According to Bruehl et al. (1996), the lengths of 95% of microvilli are less than 0.53 *μ*m for human neutrophils. Because microvilli were not modeled in our simulations, the leukocyte surface must approach the wall surface to within this length to establish tethering to endothelial cells. When a leukocyte is modeled as a nearly rigid sphere, the lift force on the leukocyte is negligible, and the leukocyte flows while sustaining a separation distance less than 0.5 *μ*m (Fig. 5D). However, if we assume even small deformability for a leukocyte, the leukocyte membrane rarely approaches the wall surface to within 0.5 *μ*m at a wall shear rate of 670/s (Fig. 4A). We also examined a lower shear rate of 330/s, which may be a representative venule shear rate (Koutsiaris et al. 2007). A separation distance of less than 0.5 *μ*m occurred under this condition, although the probability was still not large (Fig. 4B). Leukocyte rolling and adhesion are often found in venules but rarely in arterioles (House and Lipowsky 1987, 1991; Ley and Gaehtgens 1991; Perry and Granger 1991; Nazziola and House 1992), so some studies suggest that leukocyte rolling is suppressed by a low expression of adhesion molecules on endothelial cells in arterioles (Ley and Gaehtgens 1991). However, it remains unclear whether only the expression of adhesion molecules affects the number of rolling leukocytes. Our results show that the separation distance between the leukocyte and wall surfaces depends on shear rate, and that leukocytes may fail to contact the wall of straight channels at arteriole shear rate. This implies that even if the expression of adhesion molecules in arterioles is the same as in venules, a lower probability of contact at arteriole shear rate may result in a smaller number of rolling leukocytes in arterioles. Experimental studies have also reported that the number of rolling leukocytes in venules decreases with increasing shear rate (Firrel and Lipowsky 1989; Ley and Gaehtgens 1991). Reduced contacts might account for these results even in venules at relatively high shear rates.

In conclusion, leukocyte margination occurs even in large channels by the continuous passing motion of RBCs, although a longer time is needed for margination to occur in a larger channel. However, the marginated leukocytes rarely approach the wall surface to within a microvillus length at arteriole shear rate. These results for straight channels can contribute to the understanding of the physiology of leukocyte adhesion. In the real microcirculation, however, the geometry of microvessels is much more complex; microvessels do not have a smooth wall surface, and bifurcation and confluence are present. Hence, it would be interesting to study how such geometrical complexity changes leukocyte margination and contact relative to straight channels.

## Conflict of Interest

None declared.

## Figures and Tables

**Figure 7.**
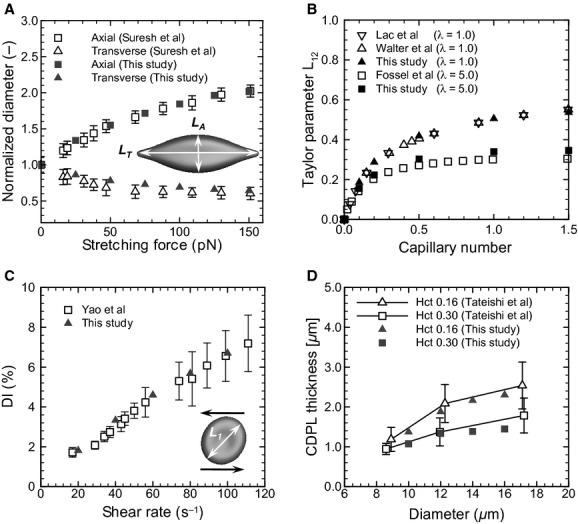
(A) Stretching of RBCs for *G*_*s*_ = 4.0 *μ*N/m. (B) The Taylor parameter of spherical cells in shear flow. (C) Deformation index of RBCs in shear flow undergoing wheel motion. (D) The thickness of the CDPL.

## Appendix

To characterize the surface shear elastic modulus for RBCs, we performed a numerical simulation reproducing the stretching of RBCs by optical tweezers (Suresh et al. 2005). We tested various values of , and the result for  = 4.0 × 10^−6^ N/m is shown in Figure A1 A. This numerical result agrees well with the experimental result of Suresh et al. (2005), and therefore this value for  was used in this paper.

We then simulated the deformation of a spherical cell (capsule) in shear flow. To compare with previous studies (Lac et al. 2004; Walter et al. 2010; Foessel et al. 2011), the Skalak constitutive law (Skalak et al. 1973) with *C *=**1 was used for the membrane, and the viscosity ratio of cytoplasm (inner fluid) to plasma (outer fluid) was set to *λ *= *μ*^in^/*μ*^out^ = 1 or *λ *= 5. The Taylor parameter *L*_12_ = (*L*_1_−*L*_2_)/(*L*_1_ + *L*_2_) is plotted as a function of capillary number  in Figure A1 B, where *L*_1_ and *L*_2_ denote the lengths of the major and minor axes of the deformed capsule in the shear plane, and *a* is the radius of the capsule. Our results are in good agreement with those presented in previous studies.

Yao et al. (2001) reported that RBCs in shear flow exhibited a wheel‐like conformation at low shear rates in their experiment. We also confirmed that RBCs gradually oriented parallel to the shear plane, and a wheel‐like motion was observed in our numerical model. They measured a deformation index, DI = (*ε*^2^−1)/(*ε*^2 ^+ 1), for *λ *= 8.48, where *ε *= *L*_1_/*L*_0_, and *L*_0_ = *d*^*R*^ is the initial diameter of the RBC. As shown in Figure A1 C, our results follow their experimental results well.

Finally, we simulated the flow of RBCs in straight circular channels to compare the thickness of CDPL with the experimental results of Tateishi et al. (1994). Figure A1 D shows that the CDPL thickness predicted by numerical simulation is again in good agreement with the experimental results.
